# Molecular nexopathies: a new paradigm of neurodegenerative disease

**DOI:** 10.1016/j.tins.2013.06.007

**Published:** 2013-10

**Authors:** Jason D. Warren, Jonathan D. Rohrer, Jonathan M. Schott, Nick C. Fox, John Hardy, Martin N. Rossor

**Affiliations:** 1 Dementia Research Centre, Department of Neurodegenerative Disease, UCL Institute of Neurology, University College London, London, UK; 2 Reta Lilla Weston Laboratories and Department of Molecular Neuroscience, UCL Institute of Neurology, University College London, London, UK

**Keywords:** neurodegeneration, dementia, neural network, nexopathy

## Abstract

Neural networks provide candidate substrates for the spread of proteinopathies causing neurodegeneration, and emerging data suggest that macroscopic signatures of network disintegration differentiate diseases. However, how do protein abnormalities produce network signatures? The answer may lie with ‘molecular nexopathies’: specific, coherent conjunctions of pathogenic protein and intrinsic network characteristics that define network signatures of neurodegenerative pathologies. Key features of the paradigm that we propose here include differential intrinsic network vulnerability to propagating protein abnormalities, in part reflecting developmental structural and functional factors; differential vulnerability of neural connection types (e.g., clustered versus distributed connections) to particular pathogenic proteins; and differential impact of molecular effects (e.g., toxic-gain-of-function versus loss-of-function) on gradients of network damage. The paradigm has implications for understanding and predicting neurodegenerative disease biology.

## Highlights

• How proteinopathies damage brain networks is a key issue in neurodegenerative disease.
• Here, we outline a solution based on the concept of ‘molecular nexopathies’.
• The concept is founded on specific interactions of network and protein properties.
• This new paradigm has far-reaching biological and clinical implications.

## Introduction

Neural networks are a key theme in contemporary neuroscience [1–3]. Operationally, a neural network can be defined as a complex system comprising nodes and links represented by neurons and their connections [4]. Neural networks extend over scales ranging from microscopic (neurons and synapses) to macroscopic (anatomical regions and fibre tracts), and may be structural (defined by physical connections; e.g., fibre tracts) or functional (defined by physiological connections). Neuroimaging techniques, such as functional MRI (fMRI) and diffusion tensor tractography [4,5], coupled with methodologies such as graph theory [2,6], have delineated intrinsic, distributed neural networks supporting cognitive functions in the healthy brain [7–9]. Neural network models have been successfully applied to common neurodegenerative syndromes [3–5,7–15], building on the key insight that neurodegenerative diseases, such as Alzheimer's disease (AD) and frontotemporal lobar degeneration (FTLD), produce distinctive clinical syndromes with regular patterns of evolution due to the spread of pathogenic protein abnormalities via large-scale brain networks.

To date, work in neurodegenerative disease has mainly focussed on linking clinical phenotypes to network alterations. However, it remains unclear how molecular (protein) abnormalities translate to network damage and, thus, clinical phenotypes; and whether pathological substrates can be predicted reliably from macroscopic network signatures. Recent advances in genetics and histopathology have enabled the detailed mapping of neurodegenerative clinico-anatomical phenotypes onto specific proteinopathies, transcending broad categories such as ‘tauopathy’ or ‘ubiquitinopathy’. Histopathological patterns of protein deposition reflect underlying molecular (biochemical or conformational) characteristics in a range of neurodegenerative diseases, most strikingly in the FTLD spectrum [16–24]. Although the concept requires further substantiation and qualification, the diffusive intercellular or ‘prion-like’ spread of pathogenic misfolded proteins holds promise as a general mechanism for the evolution of the neurodegenerative process in a wide range of diseases [8,9,25–28]. Various candidate mechanisms that might link protein pathophysiology with intercellular miscommunication and local circuit disruption have been identified [3,29,30]. However, the mechanisms that translate local effects of proteinopathies to specific patterns of large-scale network disintegration remain largely unknown.

**Figure 1:**
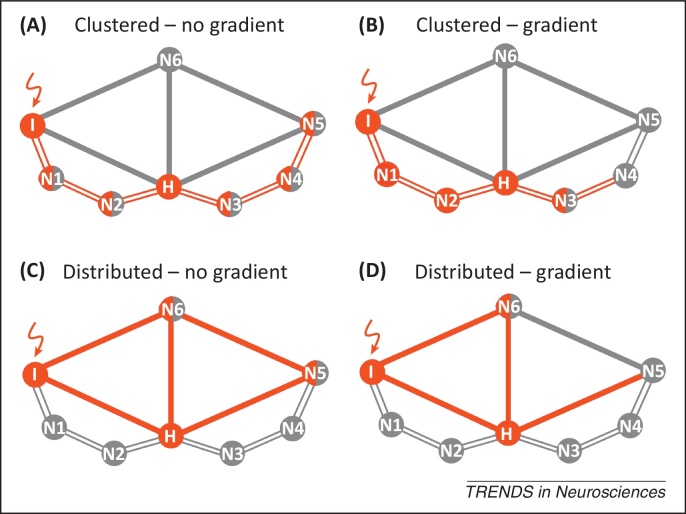
A taxonomy of molecular nexopathy mechanisms. Here, we model putative ‘templates’ of neurodegenerative network damage at a given arbitrary time point following introduction of a pathogenic protein. In each panel, the stylised local neural circuit comprises nodes (N; e.g., neuronal somas) with links (e.g., axons or dendrites) that behave as either shorter-range clustered (unfilled lines) or longer-range distributed (filled lines) connections; the most highly connected node behaves as a local hub (H), whereas node I is the site of an instigating insult (wavy arrow) associated with a pathogenic protein. Grey symbols represent unaffected or minimally affected network elements; pathogenic effects are coded in red (filled circles representing deleted network elements and half-tone circles representing dysfunctional network elements) and pathogenic effects are assumed to be potentially bidirectional across network connections. The taxonomy shown assumes two basic, interacting dichotomies arising from the conjunction of pathogenic protein and intrinsic network characteristics: selective targeting of shorter-range clustered neural connections [e.g., I–N1–N2–H–N3–N4–N5 **(A,B)**] versus longer-range distributed neural connections [e.g., I-H-N5-N6, **(C,D)**]; and effects that are relatively uniform [no gradient, **(A,C)**] or strongly graded [gradient **(B,D)**] across the network. In each case, the hub H is intrinsically relatively more vulnerable due to its high connectedness with the rest of the network [60]. Targeting of connection types could reflect subcellular compartmentalisation of pathogenic proteins and/or local synaptic properties or other morphological characteristics (e.g., targeting of dendritic versus axonal compartments). Gradients of effects could be established by intrinsic polarities in network protein expression and/or the net functional effect of a pathogenic molecular cascade (e.g., uniform toxic gain-of-function versus graded loss-of-function effects). The model that we propose requires propagation of disease effects across network elements, but does not specify the precise nature of those effects: for example, propagation could occur by direct protein transfer, protein ‘seeding’ or templating in contiguous elements, or deleterious pathophysiological signalling, all potentially operating at different stages of disease evolution. The model predicts coherence between culprit molecule, neural connection types predominantly targeted, and functional (e.g., cognitive) phenotype as the neurodegenerative process scales to the level of the whole brain (Figure 2, main text).

**Figure 2:**
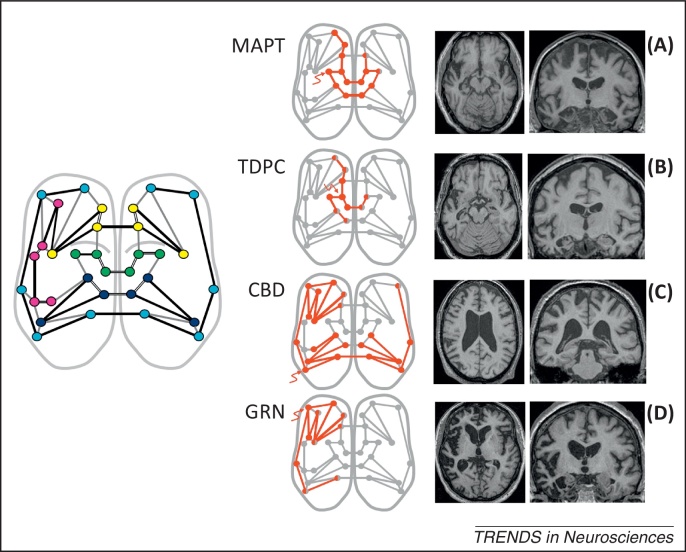
Scaling nexopathies to large-scale brain networks. The inset cartoon (left) shows a stylised axial view of the cerebral hemispheres in a normal brain. Circles represent neural network elements and colours code large-scale functional networks associated with generic clinical syndromes in previous connectivity work [7]: the anterior temporal lobe semantic network (green); the frontoinsular ‘salience’ network implicated in behavioural variant frontotemporal dementia (yellow); the dominant hemisphere speech production network implicated in progressive nonfluent aphasia (magenta); the frontoparietal network associated with corticobasal syndrome (light blue); and the temporoparietal ‘default mode’ network implicated in Alzheimer's disease (dark blue). Putative shorter-range clustered (unfilled black lines) and longer-range distributed (filled black lines) connections between network elements are shown; connections between major functional networks are also represented (grey lines). The middle panels show proposed cross-sectional schemas of network breakdown (red, following Figure 1, main text), after an instigating insult (wavy arrow) associated with a pathogenic protein. Alongside each panel, axial and coronal MRI brain sections show corresponding observed atrophy profiles in patients with representative, canonical, pathologically confirmed, proteinopathies (CBD, corticobasal degeneration associated with 4-repeat tau pathology; GRN, mutation in progranulin gene; MAPT, mutation in microtubule-associated protein tau gene; TDPC, TDP-43 type C pathology [19], associated with the clinical syndrome of semantic dementia; the left hemisphere is on the left in all sections). These atrophy profiles illustrate macroanatomical scaling of the nexopathy templates proposed in Figure 1 (main text): **(A)** predominant involvement of clustered (shorter-range) connections with uniform extension, leading to relatively focal (temporal lobe) atrophy that is relatively symmetrically distributed between the cerebral hemispheres; **(B)** predominant involvement of clustered (shorter-range) connections with a strong gradient of network damage, leading to relatively focal (temporal lobe), strongly asymmetric atrophy; **(C)** predominant involvement of distributed (longer-range) connections with uniform extension, leading to distributed, relatively symmetrical atrophy; and **(D)** predominant involvement of distributed (longer-range) connections with a gradient of network damage, leading to distributed, strongly asymmetric atrophy. A particular proteinopathy here affects network connections with particular characteristics (e.g., clustered versus distributed synaptic linkages); functional networks will be targeted according to their specific network characteristics, but the effects of a particular nexopathy will in general tend to spread between functional networks, while continuing to target connections with similar properties across these networks. This would account for empirical variability in the closeness with which proteinopathies map onto particular functional networks (e.g., the mapping is relatively close for TDPC pathology with the semantic network, whereas most proteinopathies involve the salience network). The scheme makes specific predictions about the sequence of regional involvement with particular proteinopathies (e.g., sequential involvement of homologous contralateral temporal lobe regions with TDPC pathology) (see also Figure 3, main text).

**Figure 3:**
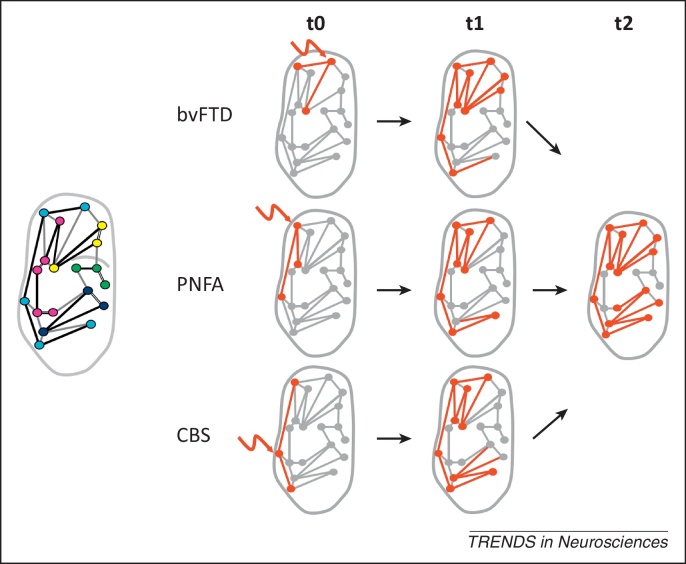
Temporal evolution of disease and phenotypic heterogeneity. This schematic illustrates the application of the molecular nexopathy concept to the problem of phenotypic heterogeneity in neurodegenerative disease, using the example of corticobasal degeneration. The inset cartoon (left) shows major functional networks in a stylised normal dominant cerebral hemisphere, colour coded as in Figure 2 (main text). The panels illustrate evolving network involvement shortly after onset of the neurodegenerative insult (t0) and at two arbitrary later time points (t1 and t2). The initial location of the insult (wavy arrow) determines the clinical presentation (behavioural variant frontotemporal dementia, bvFTD; progressive nonfluent aphasia, PNFA; or ‘classical’ corticobasal syndrome, CBS). The core corticobasal functional network (light blue in inset) is involved with disease evolution in each case; however, variable additional involvement of other contiguous functional networks (the salience network, speech production network or default mode network) modulates the phenotype. Each of these phenotypes arises from a common template of network involvement determined by the type of neural connection predominantly involved (here, represented as longer-range distributed intrahemispheric projections); the common nexopathy signature of corticobasal degeneration is revealed in the temporal profile of disease evolution.

Here, we address this problem. We propose the term ‘molecular nexopathy’ (Latin *nectere*, tie) to refer to a coherent conjunction of pathogenic protein and intrinsic neural network characteristics expressed as a macroanatomical signature of brain network disintegration. We argue that improved understanding of the molecular mechanisms of network disintegration will constitute a new paradigm of neurodegenerative disease. The essential features of the paradigm that we propose are presented in Box 1 and Figures 1–3. We now consider potential mechanisms whereby molecular dysfunction might be linked to neural circuit disruption. We then assess the extent to which the molecular nexopathy paradigm can be reconciled with the central problems of disease evolution and phenotypic heterogeneity, and propose experimental tests of the paradigm in future work.

## How do pathogenic molecules produce specific brain network disintegration?

### Networks show variable intrinsic vulnerability to proteinopathies

The molecular nexopathy paradigm makes no assumptions about the instigating event that triggers the neurodegenerative process, which might be stochastic but which results in the creation of a potentially pathogenic molecule. However, once initiated, the topography of neurodegeneration preferentially targets network elements that are vulnerable to the instigating molecular species (Figure 1). Emerging evidence, including inoculation experiments in animals [31–33] (Box 1), implies that a neurodegenerative process may ‘home’ to a brain region (or regions) based on intrinsic vulnerability to the pathogenic protein. Neurodegeneration may propagate by ‘prion-like’ seeding or templating of the protein abnormality (e.g., conformational misfolding) across neural connections, in addition to physical transfer of instigating pathogenic proteins. The presence of a specific pathogenic abnormality that propagates across a network would distinguish a neurodegenerative molecular nexopathy from other diseases that disrupt brain networks (for example, stroke and traumatic brain injury): one important corollary is that compensatory or homeostatic responses are ultimately inadequate in neurodegenerative nexopathies.

Regional neural vulnerability to a proteinopathy could reflect anatomically restricted expression of the culprit protein by cell populations or additional epigenetic factors that direct the expression of the protein to particular brain areas [34–36] or determine neuronal susceptibility to toxic events [10]. Regional differentiation of protein expression is a fundamental feature of normal brain development [37], establishing intrinsic specificities of connections within neural circuits [38,39] and thereby in turn directing the function of the circuit. Therefore, local profiles of protein expression could confer selective vulnerability or resistance of particular network elements to particular neurodegenerative diseases, and would also help drive the functional phenotypic signature of the disease. For example, differential expression of neuroprotective factors has been linked to the relative vulnerability of particular neuronal populations in the basal ganglia in Parkinson's disease [40], and regional expression of genes involved in inflammatory signalling may modulate disease onset with progranulin (GRN) mutations [41]. By contrast, epigenetic effects during brain development alter the regulation and expression of amyloid precursor protein and potentially influence the later development of AD [42]. Brain areas that are highly neuroplastic, more specialised, or phylogenetically more recent (for example, the language system) may be relatively more vulnerable to proteinopathies [43], whereas primary motor and sensory cortex both show relative resistance. The process of neurodegeneration might selectively ‘unravel’ the sequence of normal ontogeny within the vulnerable network (for example, by withdrawing essential trophic support, repair mechanisms, or physiological signalling across damaged synaptic connections) [29].

Regional specificity might also arise from cellular morphological factors, particularly at synaptic connections: even if a protein is widely expressed in the brain, its effects may propagate only in particular cell types [44] or across specific patterns of connections [29]. Animal models have demonstrated exquisite microanatomical and biochemical specificity of intercellular connections in key vulnerable structures (such as hippocampus) [45,46]. Age-related neuronal resprouting may enhance local deposition of amyloid precursor protein in entorhinal cortex early during the course of AD [47]. Protein expression and morphological specificity at receptors and synapses would interact with subcellular molecular factors. For example, tau isoforms show distinctive subcellular distributions [48,49]. Deranged microtubular transport of abnormal tau facilitates accumulation of aggregated tau in somatic and dendritic rather than axonal compartments [50,51], whereas diffusible tau may further focus pathogenic effects of the protein on local synaptic and glial connections [51]. The role of glial elements is poorly understood, but may influence local expression and development of network damage, reflected macroscopically in the relative extent of grey matter versus white matter damage [52].

### Proteinopathies are fitted to neural circuits

Central to the molecular nexopathy paradigm is the ‘fit’ between the pathogenic molecule and local neural circuit (and, ultimately, large-scale network) characteristics (Figures 1 and 2). Although data on specific local interactions of neural circuits with proteins remain limited, a proteinopathy might spread between brain regions by causing connected regions to develop the same intracellular protein abnormality or, less directly, by affecting the function of those connected regions [3]. The coupling between functional and structural connectivity in neurodegenerative diseases remains poorly defined: however, initial dysfunction could promote subsequent molecular alterations and destruction of network elements, as shown in lesion and tract-tracing studies in humans and nonhuman primates [5,6,53]. In more theoretical terms, the effects of a proteinopathy on a network might be regarded as a form of ‘information’ flow, where information signifies a change in network function (in particular, alterations in synaptic properties) associated with the introduction of the abnormal protein [3]. The characteristics of information exchange across artificial neural networks have attracted considerable interest in computational neurobiology [2,54]: this theoretical framework might be adapted to the case of proteinopathies. Pathogenic molecular effects often have time constants that are much longer than those typically associated with information flow in neural networks. However, dynamic downstream alterations in synaptic function across circuits would occur over much shorter timescales. A further, potentially related, factor is the role of pervasive patterns of activity in circuit function and in predisposing networks to the effects of neurodegenerative disease [55]: examples include the differential and possibly use-dependent susceptibility of particular motor pools to amyotrophic lateral sclerosis (ALS) [27], or the altered trafficking of amyloid and tau in the isodendritic core associated with perturbations of the sleep–wake cycle in AD [56]. Putative behavioural and activity-related factors are likely to interact with underlying genetic and epigenetic predisposing factors, and the causal sequence in general remains to be determined.

An important theoretical motivation for applying the network information-processing framework to the neurodegenerative proteinopathies is the rich taxonomy of network activity patterns that follow from relatively simple starting assumptions when modelling the evolution of artificial neural networks [54]. In particular, it has been shown in such artificial networks that patterns of neural activity in local network elements can, under certain conditions, scale up to the entire network; that the characteristics of local microcircuits strongly influence the activity pattern produced by the network as a whole; and, furthermore, that these patterns may be highly polarised. All these are network properties predicted in the case of the neurodegenerative proteinopathies, for which an ‘activity pattern’ could be interpreted as the cumulative effect of protein-associated network damage integrated over time [55].

Relevant network and protein characteristics have yet to be worked out in detail for neurodegenerative proteinopathies. However, it has been shown that brain networks have ‘small-world’ properties expressed as a high degree of clustering among network elements and short average path lengths between clusters [2]. These small-world properties suggest a basic dichotomy between shorter-range neural connections within clusters (local neural circuits, with relatively long neural path length) and relatively sparse longer-range neural connections (with relatively short neural path length) between clusters, in line with highly segregated and hierarchical brain network architectures observed empirically [6] and with limited evidence concerning the cellular pathophysiology of certain proteinopathies [48,51,57,58] (Box 1). This putative dichotomy between clustered and distributed network connections suggests a morphological basis for partitioning neurodegenerative diseases according to whether they produce relatively localised versus more distributed profiles of macroscopic brain atrophy [18,20] (Figures 1 and 2). ‘Clustered’ and ‘distributed’ connections could be modelled in synthetic neural circuits and could be defined in brain networks using anatomical methods that can measure effective path lengths between network elements [6]; for example, dynamic causal modelling. Alternative morphological dichotomies might also operate: for example, selective targeting of excitatory versus inhibitory projections [44].

The degree of macroanatomical asymmetry of network damage within and between cerebral hemispheres appears to be a further partitioning characteristic of many neurodegenerative diseases (Figure 2), although to demonstrate interhemispheric asymmetries, it may be necessary to retain individual hemispheric asymmetry profiles when pooling data in group neuroimaging studies [18,20–22]. Network asymmetries could be determined, in part, by configurational features of the host network that might tend to polarise network activity. Such polarity has been demonstrated in a computational model of Huntington's disease [44]. Asymmetries might be predicted based on extrapolation from network architectures in the brains of other species. For example, the nodes of large-scale networks in the macaque brain have a highly nonuniform distribution within the cortex and the connections between nodes are hierarchically organised [6]. Given that the human brain shares network homologies with that of the macaque [53], this architecture would tend to focus the effects of neurodegenerative disease at particular vulnerable ‘hub’ regions (for example, in prefrontal cortex and posterior cingulate–precuneus [1,59]). Much more generally, it has been shown that highly connected network elements are intrinsically more vulnerable to extinction following perturbing events in a variety of hierarchical systems, ranging from ecology to economics [60]. In the context of neurodegenerative disease, ‘extinction’ might be equated to destruction of highly connected network elements after introduction of a pathogenic protein and susceptibility from connectedness might, for example, predict disproportionate vulnerability of dominant hemisphere language hubs in the progressive aphasias [43] and medial parietal hubs binding the default mode network in AD [1,4,15,55,59]. If functional connections between brain regions are defined based on the strength and direction of spontaneous activity correlations, fMRI data suggest a fundamental dichotomy between ‘positive’ connections that are dominant within a cerebral hemisphere versus ‘negative’ connections that are dominant between hemispheres [61]: negative interhemispheric functional correlations will tend to establish intrinsically asymmetric interhemispheric interactions that could be exploited by neurodegenerative pathologies (Figure 2).

It is unlikely *a priori* that any set of neuronal or neural network features would confer vulnerability uniquely to a single molecular species. However, further specificity in the profile of network involvement (in particular, whether strong polarity of damage is expressed across the brain) may be driven, in part, by functional characteristics of the pathogenic protein itself.

### Directional protein dysfunction drives network asymmetries

Across the spectrum of potentially pathogenic proteins, there is a basic distinction between toxic-gain-of-function (deleterious effects of protein accumulation) and loss-of-function (impaired physiological, signalling or trophic) molecular effects [57,62]. The loss of function of a key protein is likely to lead ultimately to the loss of function of the affected network element and, therefore, might be regarded in computational terms as ‘inhibiting’ the affected element; the net computational effect of a toxic gain of function is more difficult to predict. Large-scale network asymmetries (i.e., asymmetric macroscopic atrophy profiles) might result from interaction of intrinsic connectivity structure with a gradient of molecular effects across the vulnerable network.

We envisage that, within an affected network, an overall toxic gain of function will spread relatively uniformly, whereas an overall loss-of-function effect will establish a gradient of tissue loss due to attenuation of ‘downstream’ synaptic inputs. Such polarising network-level effects of loss-of-function proteinopathies would be in line with a net ‘inhibitory’ action on damaged connections, because selective inhibition of network elements can generate highly polarised network structures and self-amplifying network activity patterns in computational models [54,61,63,64]. Proteinopathic effects would interact with (and may, in part, be driven by) intrinsic, ontogenetic network gradients [38,39]. Trophic effects modulate intercellular gradients in normal morphogenesis and developmental disorders [65] as well as in computational models [66]. Certain loss-of-function effects could become self amplifying due to additional, ‘catastrophic’ mechanisms that might be specific to particular protein alterations: an example is GRN mutations, which may inhibit neuronal repair processes leading to accelerated collapse of network architecture [67].

Although it is unlikely that polarised protein effects operate in pure form in the brain [57,62], for a given disease process and disease stage, toxic gain-of-function or loss-of-function effects may dominate at the network level (Figure 1). Intracellularly, particular pathogenic proteins have complementary loss-of-function and toxic-gain-of-function effects [62]. However, the overall primary balance of those effects across a neural network may depend on specific molecular actions at key network elements (e.g., synapses) that act as the final common pathway for network damage. Additional specificity may be conferred by biochemical characteristics and conformational signatures of protein subtypes within broad categories, such as tau and Tar DNA-binding protein 43 (TDP-43) [24,49]. We currently lack such specific information for most key pathogenic proteins in the neurodegenerative spectrum [62]. There is further substantial potential for interactions among pathogenic proteins (for example, between tau and beta-amyloid in AD [28]). Protein-specific effects might modulate intrinsic network connectivity properties, contributing to phenotypic variation associated with particular proteins within a common network architecture [for example, the relatively symmetric atrophy profile associated with microtubule-associated protein tau (MAPT) mutations versus the strongly asymmetric profile associated with TDP-43 type C (TDPC) pathology [19] within anterior temporal lobe networks [18]].

## Temporal evolution and the problem of heterogeneity

A critical feature of neurodegenerative molecular nexopathies is likely to be their pattern of evolution in time as well as spatially within the brain. The rapidity of network breakdown might depend on the relative proportions of connection types affected by the pathological process, the predominant involvement of longer-range connections corresponding to rapid spread and involvement of clustered connections corresponding to slower spread, respectively. This would fit with available data for certain neurodegenerative disorders. For example, patients with MAPT mutations and relatively focal anterior temporal lobe damage have, on average, slower rates of overall brain atrophy and survive substantially longer compared with patients with GRN mutations associated with widespread intrahemispheric damage [68]; interhemispheric asymmetry increases with advancing disease in association with GRN mutations [17]; but MAPT and GRN mutations produce similar local rates of atrophy within key structures such as the hippocampus [69]. Taken together, such evidence suggests that disease effects are preferentially amplified if long intrahemispheric fibre tracts are implicated.

The temporal evolution of atrophy profiles associated with a particular proteinopathy may reveal a characteristic signature of network involvement that unites apparently disparate phenotypes (Figure 3). For example, tauopathies in the FTLD spectrum (such as corticobasal degeneration) may present with a behavioural syndrome due to frontal lobe involvement, with a language syndrome due to involvement of peri-Sylvian cortices in the dominant hemisphere, with a parietal lobe syndrome or with atypical parkinsonism: the nexopathy paradigm predicts phenotypic convergence over time due to progressive erosion of core frontoparietal, frontotemporal, or frontosubcortical networks implicated in particular tauopathies [18]. There is substantial evidence for such phenotypic convergence in the FTLD spectrum [18,70] and related overlap syndromes such as FTD-ALS [71]; however, precise correlations with particular brain networks have yet to be widely established. Similarly, variant AD phenotypes have been interpreted as modulating a core temporoparietal-prefrontal ‘default mode’ network [15]. Phenotypic convergence implies that initial stochastic insults anywhere in a vulnerable network will lead ultimately to a common signature of network breakdown, although the precise sequence of network involvement will tend to reflect the initial locus of pathology within the network (Figure 3).

This concept of the differential involvement of a core vulnerable network (with increasingly complete involvement of the network over time) suggests one possible solution to the apparent paradox of individual phenotypic variation associated with particular proteinopathies [70]. The clinico-anatomical expression of a given proteinopathy often varies between individuals as well as between syndromic subgroups [15,27,43]. The molecular nexopathy paradigm requires that the neuroanatomical profile of disease evolution is not random, but adheres to a spatiotemporal ‘template’ of network damage: the location of disease onset within the vulnerable network may vary between individuals, but progression of the particular disease in individuals, over time, would tend to recapitulate a characteristic pattern of network involvement. Therefore, to establish the disease template conclusively will entail detailed natural history studies: such studies in ALS have exploited the well-understood and highly regular organisation of the cerebral motor pools and their connections [27,71,72]. This example has also underlined the considerable functional reserve inherent in many brain networks, implying that ‘noisy’ information transfer by surviving elements can support network functions until a critical stage of network failure is reached [3,10]. Both neuroimaging and behavioural metrics will be required to capture the ‘prodromal’ phase of early network alterations as well as compensatory or homeostatic responses [3,11,73].

The effects of a particular proteinopathy need not and generally will not be restricted to a single vulnerable large-scale network (Figures 2 and 3). Rather, ‘nexopathy’ inheres in the type of network connections affected. To the extent that connections with particular properties are concentrated in a single functional network, the nexopathy paradigm would predict that proteinopathies targeting those connections should principally affect that network: this may explain the existence of neurodegenerative diseases (such as those associated with MAPT mutation and TDP-C pathology) that preferentially target the anterior temporal–inferior frontal lobe semantic network [7,18,21,69,74,75]. In general, however, connection types will be represented in more than one functional network (Figure 2), providing a mechanism for the spread of proteinopathies between networks, with further phenotypic variation and potential overlap of clinico-anatomical profiles among proteinopathies [18,70]. Functional interactions between large-scale brain networks will also tend to obscure network specificities [76]. Ultimately, disease spread via secondarily connected systems throughout the brain implies that network and connection specificity will be most evident earlier during the evolution of a particular disease.

As a final important caveat on the differentiation of nexopathies, it is unlikely that complete specificity will apply across the entire gamut of pathogenic proteins implicated in neurodegenerative disease. Rather, we envisage a taxonomy of predictable profiles of network disintegration: within the taxonomy, particular profiles of nexopathy might be common to different pathogenic proteins to the extent that those proteins share key properties that promote network damage or dysfunction. Different proteins might, for example, participate in a common, multicomponent pathogenic cascade (perhaps best characterised at present for AD [15,57]).

## Future directions: testing the molecular nexopathy paradigm

The molecular nexopathy paradigm requires substantiation drawing on diverse molecular, cellular, and systems neuroscience (behavioural and neuroimaging) approaches, including synthetic and *in vitro* neural circuits, transgenic and other animal models, and dynamic macroanatomical techniques [12]. Clinical studies will continue to have a key role in delineating the sometimes counterintuitive phenotypes that define brain network disintegration (Figure 2); and phenotyping should be supported by detailed correlative histological studies. If the molecular nexopathy concept can be substantiated, it would hold great potential for understanding, tracking, and predicting the expression of neurodegenerative proteinopathies. Indeed, the concept need not be restricted to proteins: for example, abnormal cellular signalling linked to carbohydrate moieties could in principle give rise to ‘sugar nexopathies’ [77]. Several specific, testable questions follow (Box 2) that collectively could direct future work. Beyond the principled evaluation and monitoring of candidate therapies, if mapping network breakdown is equivalent to mapping the expression of a molecular lesion, then delineating such a network could be regarded as a direct ‘*in vivo* assay’ of the function of the protein: a concept analogous to that proposed for the theoretical neural nets of computational neuroscience [78]. If neural network dysfunction were sufficiently well specified, this could in turn help identify (or discriminate between) candidate molecular mechanisms driving the neurodegenerative process and suggest rational candidate therapies.

## Box 1 The molecular nexopathy paradigm: key substrates and constraints

### Molecular, microstructural, and functional substrates

Pathogenic proteins, including misfolded aggregates and toxic oligomers, could promote neural network disintegration by disrupting synaptic function or maintenance [57], axonal transport or repair [79], or via ‘downstream’ trophic [58,80] or cell–cell signalling abnormalities [3,10,29,62,81–85]. Disease effects would exploit intrinsic network vulnerabilities: in particular, developmental patterns of protein expression and structural and functional interactions across neural circuits [38,39] (Figure 1, main text). Shorter-range or clustered dendritic and interneuronal connections appear particularly vulnerable to some tauopathies [48,51], whereas longer-range, more widely distributed (axonal) projections may be relatively more vulnerable to other molecular insults (e.g., toxic oligomers derived from amyloid precursor protein [58] or deficiency of trophic or protective factors such as GRN [57]). The balance between molecular net toxic gain-of-function and loss-of-function effects [57,62] might help to determine the extent to which proteinopathies exert uniform or graded effects across neural circuits (Figure 1, main text). ‘Soft-wired’ alterations in synaptic function, in part reflecting pervasive network activity patterns, might give way to subsequent (irreversible) ‘hard-wired’ structural damage and cell loss.

### Disease propagation

Transcellular and, in particular, synaptically mediated diffusion of misfolded proteins and ‘permissive templating’ of protein misfolding appear to be general principles of disease evolution across a range of neurodegenerative proteinopathies [26,86], including prion [85], beta-amyloid [31–33], tau [87–90], alpha-synuclein [91,92], TDP-43 [71,72], and superoxide dismutase 1 [27]. Inoculation with beta-amyloid and tau triggers uptake by cells and ‘templated’ conformational alteration of normally folded protein to a potentially pathogenic, misfolded state [26,86]. Trans-synaptic spread of tau-containing tangle pathology from entorhinal cortex neurons occurs in transgenic mice that only express mutant MAPT in those neurons [89,90].

### Macroanatomical signatures

Particular structural and functional neuroimaging profiles of network disintegration have specific molecular associations [7,16,17,20–23,68,69,74,75,93–95] (Figure 2, main text). In the FTLD spectrum, structural neuroimaging evidence suggests a scheme for partitioning pathologies according to whether the macroanatomical brain atrophy profile is localised or distributed, and whether atrophy is relatively symmetric or strongly asymmetric between the cerebral hemispheres [18,23] (Figure 2, main text). In the case of AD, early (even presymptomatic) functional and structural alterations occur within a specific distributed parieto temporofrontal network mediating ‘default mode processing’ or stimulus-independent thought in healthy individuals [4,11] and network disintegration tracks pathological disease staging [11,14,96]. Variant AD phenotypes, such as posterior cortical atrophy and logopenic progressive aphasia, may reflect differential involvement of corticocortical projection zones that together constitute a distributed AD-vulnerable network [15,97], although the precise pathophysiological roles of the two major candidate pathogenic proteins (beta-amyloid and phosphorylated tau) and the factors that modulate the profile of network damage remain contentious [4,11,15]. Convergence of syndromes as disease evolves may hold a solution to the problem of phenotypic heterogeneity (Figure 3, main text).

## Box 2 Testing the paradigm: outstanding questions and future directions

### Is diffusive protein spread a general mechanism of neurodegeneration?

The strength of evidence for ‘prion-like’ spread, permissive templating, and direct cell–cell transmission varies among proteinopathies, and has not been established for some (e.g., fused-in-sarcoma protein) [98]. Protein spread could occur via mechanisms other than network pathways (e.g., extracellular diffusion [26,27]). Nonprion diseases have not been shown to have prion-like spontaneous infectivity and, typically, evolve more slowly [86].

*Hypothesis*

Diffusive protein spread is a general mechanism of network disintegration.

*Key experiments*

Inoculation and tracer studies in animal models [31–33] and the development of novel molecular model systems [77].

### How do protein properties relate to profiles of network degeneration?

Protein properties and expression profiles remain to be related in detail to cell morphological features, dynamics of cellular transport mechanisms, and intercellular interactions. For various pathogenic proteins, the final net ‘direction’ of effects (loss-of-function versus toxic gain-of-function effects) remains contentious [62]. For several neurodegenerative diseases, the pathogenic protein species or the pathogenic form of the protein has not been unambiguously determined [25].

*Hypothesis*

Network degeneration arises predictably from interactions of pathogenic protein properties with specific network morphological and functional characteristics.

*Key experiments*

Computational modelling of artificial neural networks with biologically plausible properties [44]; development of model neural circuits *in vitro* [99] and *in vivo* [38,39]; histomorphometric and immunolabelling analyses of subcellular protein targets and putative connection vulnerabilities (e.g., dendritic versus axonal) [48,51]; correlative phenotypic-histological studies; and cognitive science paradigms to characterise network activity and architectures [27,30,100].

### Are empirical patterns of disease evolution consistent with nexopathy models?

Certain proteinopathies have specific macroanatomical signatures of network disintegration [4,7,11,13–18,20–23], but disease overlap and heterogeneity are substantial. The problem of heterogeneity may be resolved, in part, by mapping longitudinal profiles of disease evolution, using neuroimaging tools that can capture convergent structural and/or functional changes across the brain and over time [7,8,11]. The molecular nexopathy paradigm makes specific predictions about the sequence of regional evolution with particular proteinopathies (Figures 2 and 3, main text). fMRI reflects synaptic function and can assess functional connectivity of networks when active and at rest [1,7], including potentially compensatory and homeostatic responses [3,10,73]; in addition, diffusion tensor imaging can assess structural connectivity of white matter pathways that bind large-scale networks [4,5].

*Hypothesis*

Macroanatomical spatiotemporal network signatures arise predictably from underlying molecular lesions.

*Key experiments*

Longitudinal quantitative tracking of regional tissue damage in different neurodegenerative diseases in large patient cohorts (in particular, those with defined molecular lesions) derived from multicentre collaborative platforms [101]; connectivity metrics (e.g., dynamic causal modelling) to assess relations between network elements (e.g., putative shorter- versus longer-range connections) and dynamic, physiologically motivated techniques, such as magnetoencephalography (MEG) [12]; development of new molecular ligands (e.g., phosphorylated tau); and practical quantitative metrics of network function and connectivity [1,2].

### What are the implications for disease diagnosis, prognosis, and treatment?

Besides predicting underlying proteinopathies, improved understanding of determinants of network disintegration would enable the prediction and more accurate tracking of disease evolution. Identification of specific neural network dysfunction pre-dating the loss of structural integrity may enable modulation of culprit molecular deficits. Specific molecular treatments could be directed at modifying protein mechanisms that sustain network integrity [102] or bolstering inhibitory processes to counteract excitotoxic effects [103]. Interventions that reduce local protein concentrations might ameliorate diffusive protein spread for many diseases.

*Hypothesis*

Profiles of network disintegration provide biomarkers for tracking disease evolution and treatment response.

*Key experiments*

Detailed natural history studies and incorporation of network-level metrics in clinical trials.
